# An Integrated Fuzzy Approach for Strategic Alliance Partner Selection in Third-Party Logistics

**DOI:** 10.1100/2012/486306

**Published:** 2012-12-05

**Authors:** Burak Erkayman, Emin Gundogar, Aysegul Yılmaz

**Affiliations:** Department of Industrial Engineering, Sakarya University, 54187 Adapazarı, Sakarya, Turkey

## Abstract

Outsourcing some of the logistic activities is a useful strategy for companies in recent years. This makes it possible for firms to concentrate on their main issues and processes and presents facility to improve logistics performance, to reduce costs, and to improve quality. Therefore provider selection and evaluation in third-party logistics become important activities for companies. Making a strategic decision like this is significantly hard and crucial. In this study we proposed a fuzzy multicriteria decision making (MCDM) approach to effectively select the most appropriate provider. First we identify the provider selection criteria and build the hierarchical structure of decision model. After building the hierarchical structure we determined the selection criteria weights by using fuzzy analytical hierarchy process (AHP) technique. Then we applied fuzzy technique for order preference by similarity to ideal solution (TOPSIS) to obtain final rankings for providers. And finally an illustrative example is also given to demonstrate the effectiveness of the proposed model.

## 1. Introduction

Supply chain management involves the design and management of seamless, value-added processes across organizational boundaries to meet the real needs of the end customer [1–3]. Logistics play a significant role in integrating the supply chain of industries. However, as the market becomes more global, logistics are now seen as an important area where industries can cut costs and improve their customer service quality [4]. Logistics outsourcing and third-party logistics originated in the 1980s as important means of improving supply chain effectiveness [5]. 

Estimates indicate that the proportion of companies in the US implementing this approach has increased by 5–8% annually between 1996 and 2004 [6]. Moreover, in 2005 no less than 80% of the Fortune 500 Companies stated that they relied on TPL [7]. Current predictions indicate growth rates in the range of 15–20% between 2009 and 2011 in both Western Europe and the USA [8, 9].

Third it can be defined as a managed process of transferring activities to be performed by others. Logistics outsourcing or third party logistics (3PL) involves the use of external companies to perform logistics functions that have traditionally been performed within an organization [10]. Outsourcing can be a value-enhancing activity. However, the top benefits for companies outsourcing are often related to costs savings [11, 12]. Logistics outsourcing or third-party logistics (3PL) is an emerging trend in the global market. Basically, a 3PL provider (hereinafter referred to as provider) involves using external companies to perform logistics functions which have been conventionally operational within an organization [13]. 

Outsourcing involves the procurement of physical and/or service inputs from outside organizations either through cessation of an activity that was previously performed internally or abstention from an activity that is well within the capability of the firm [14]. The main benefits of logistics alliances are to allow the outsourcing company to concentrate on the core competence, increase the efficiency, improve the service, reduce the transportation cost, restructure the supply chains, and establish the marketplace legitimacy [15–17].

Finding the right partner requires careful screening and can be a time-consuming process. Developing an understanding of partners' expectations and objectives can also take time [18].

Multiple criteria decision-making (MCDM) is a powerful tool widely used for evaluating problems containing multiple, usually conflicting criteria [19]. In this study we defined the provider selection problem as MCDM problem, and proposed a fuzzy approach to solve it. Decision making problems subject to subjective evaluations must be considered in fuzzy environment. Because of this situation application of fuzzy MCDM approaches is preferred.

The proposed method integrates fuzzy AHP and fuzzy TOPSIS techniques for provider selection that satisfies the needs of Third-Party Logistics company. First, the weights of criteria have been calculated using fuzzy AHP, and fuzzy TOPSIS is used for the selection of providers.

The remainder of the study is arranged as follows: Section 2 briefly describes the proposed methods.  Section 3 describes the proposed model. An illustrative example is given in Section 4. In Section 5, results and suggestions are discussed.

## 2. Methods

### 2.1. The Fuzzy AHP Method

AHP [20] is one of the most extensively used MCDM analysis tools for modeling the unstructured problems in different areas such as politics, economic, social, and management sciences. AHP assumes that evaluation criteria can be completely expressed in a hierarchical structure. The data acquired from the decision-makers are pairwise comparisons concerning the relative importance of each of the criteria, or the degree of preference of one factor to another with respect to each criterion. In the conventional AHP, the pairwise comparison is made by using a ratio scale. Even though the discrete scale has the advantages of simplicity and ease of use, it does not take into account the uncertainty associated with the mapping of one's perception (or judgment) to a number. In order to deal with the uncertainty and vagueness from the subjective perception and the experience of human in the decision-making process, many fuzzy AHP methods are proposed by various authors [18].

In this study we use the Chang's extent analysis method for fuzzy AHP. According to Chang [21], let *X* = {*x*_1_, *x*_2_,…, *x*_*n*_} be an object set, and *U* = {*u*_1_, *u*_2_,…, *u*_*m*_} be a goal set. According to the method of Chang's [21] extent analysis, each object is taken and extent analysis for each goal is performed, respectively. Therefore, *m* extent analysis values for each object can be obtained, with the following signs:

(1)   Mgi1,Mgi2,…,Mgim, i=1,2,…,n,

where all the *M*_*g*_*i*__^*j*^  (*j* = 1,2,…, *m*) are triangular fuzzy numbers.

The value of fuzzy synthetic extent with respect to the *i*th object is defined as

(2) $$\begin{array}{c} S_{i}=\sum_{j=1}^{m}M_{g_{i}}^{j}⊗{\left[\sum_{i=1}^{n}\sum_{j=1}^{m}M_{g_{i}}^{j}\right]}^{-1}. \end{array}$$

The degree of possibility of *M*_1_ ≥ *M*_2_ is defined as

(3) $$\begin{array}{c} V\left(M_{1}\geq M_{2}\right)=\underset{x\geq y}{\sup}\left\lfloor \min\left(\mu_{M_{1}}\left(x\right),\mu_{M_{2}}\left(y\right)\right)\right\rfloor . \end{array}$$

When a pair (*x*, *y*) exists such that *x* ≥ *y* and *μ*_*M*_1__(*x*) = *μ*_*M*_2__(*y*) then we have *V*(*M*_1_ ≥ *M*_2_) = 1. Since *M*_1_ and *M*_2_ are convex fuzzy numbers we have

(4) V(M1≥M2)=1 if  m1≥m2,V(M1≥M2)=hgt(M1∩M2)=μM1(d),

where *d* is the ordinate of the highest intersection point *D* between *μ*_*M*_1__ and *μ*_*M*_2__.

When *M*_1_ = (*l*_1_, *m*_1_, *u*_1_) and *M*_2_ = (*l*_2_, *m*_2_, *u*_2_), the ordinate of *D* is given by

(5) V(M2≥M1)=hgt(M1∩M2)=μM2(d)={1m2≥m1,0l1≥u2,l1−u2(m2−u2)−(m1−l1)otherwise.

To compare *M*_1_ and *M*_2_, we need both the values of *V*(*M*_1_ ≥ *M*_2_) and *V*(*M*_2_ ≥ *M*_1_) and the intersection between *M*_1_ and *M*_2_.

The degree possibility for a convex fuzzy number to be greater than *k* convex fuzzy numbers *M*_*i*_  (*i* = 1,2,…, *k*) can be defined by

(6) V(M≥M1,M2,…,Mk)  =V[(M≥M1),(M≥M2),…,(M≥Mk)]  =min⁡V(M≥Mi), i=1,2,…,k.

Assume that

(7) $$\begin{array}{c} d^{\prime }\left(A_{i}\right)=\min V\left(S_{i}\geq S_{k}\right). \end{array}$$

For *k* = 1,2,…, *n*; *k* ≠ *i*. Then the weight vector is given by

(8) $$\begin{array}{c} W^{\prime }={\left(d^{\prime }\left(A_{1}\right),d^{\prime }\left(A_{2}\right),\ldots ,d^{\prime }\left(A_{n}\right)\right)}^{T}, \end{array}$$

where *A*_*i*_ (*i* = 1,2,…, *n*) are *n* elements.

Via normalization, the normalized weight vectors are

(9) $$\begin{array}{c} W={\left(d\left(A_{1}\right),d\left(A_{2}\right),\ldots ,d\left(A_{n}\right)\right)}^{T}, \end{array}$$

where *W* is a nonfuzzy number.

### 2.2. The Fuzzy TOPSIS Method

The TOPSIS [22] is widely used for tackling ranking problems in real situations. Despite its popularity and simplicity in concept, this method is often criticized for its inability to adequately handle the inherent uncertainty and imprecision associated with the mapping of the decision-maker's perception to crisp values. In the traditional formulation of the TOPSIS, personal judgments are represented with crisp values. However, in many practical cases the human preference model is uncertain and decision-makers might be reluctant or unable to assign crisp values to the comparison judgments [23]. Having to use crisp values is one of the problematic points in the crisp evaluation process. One reason is that decision-makers usually feel more confident to give interval judgments rather than expressing their judgments in the form of single numeric values. As some criteria are difficult to measure by crisp values, they are usually neglected during the evaluation. Another reason is mathematical models that are based on crisp value. These methods cannot deal with decision-makers' ambiguities, uncertainties, and vagueness which cannot be handled by crisp values [24]. The use of fuzzy set theory [25] allows the decision-makers to incorporate unquantifiable information, incomplete information, non-obtainable information, and partially ignorant facts into decision model [26]. 

TFNs appear to be a valid tool, offering a well balanced compromise between computational costs and accuracy in the final ranking [27].

The steps of fuzzy TOPSIS are as follows [28, 29].

**Step 1**

Choose the appropriate linguistic variables for the alternatives with respect to criteria. The linguistic variables are described by TFNs, such as ${\tilde{x}}_{ij}=(a_{ij},b_{ij},c_{ij})$.

**Step 2**

Construct the fuzzy decision matrix and the normalized fuzzy decision matrix:

(10) $$\begin{array}{c} \tilde{R}={\left[{\tilde{r}}_{ij}\right]}_{m\times n}. \end{array}$$

**Step 3**

Calculate the weighted normalized fuzzy decision matrix. The weighted normalized value ${\tilde{v}}_{ij}$ is calculated as

(11) $$\begin{array}{c} \tilde{V}={\left[{\tilde{v}}_{ij}\right]}_{n\times J},\;i=1,2,\ldots ,n,\;\;j=1,2,\ldots ,J. \end{array}$$

**Step 4**

Identify positive-ideal (A*) and negative ideal (*A* ^−^) solutions. The fuzzy positive-ideal solution (FPIS, A*) and the fuzzy negative-ideal solution (FNIS, *A* ^−^) are shown in the following equations:

(12) $$\begin{array}{c} A^{*}=\left\{{{\tilde{v}}_{1}}^{*},{{\tilde{v}}_{2}}^{*},\ldots ,{{\tilde{v}}_{i}}^{*}\right\},\\ A^{-}=\left\{{{\tilde{v}}_{1}}^{-},{{\tilde{v}}_{2}}^{-},\ldots ,{{\tilde{v}}_{i}}^{-}\right\}, \end{array}$$

where ${{\tilde{v}}_{ij}}^{*}=w_{j}⊗\left(1,1,1\right)$, ${{\tilde{v}}_{ij}}^{-}=w_{j}⊗(0,0,0)$ for all *j* = 1,2,…, *n*.

**Step 5**

Calculate the distance of each alternative from A* and *A* ^−^ using following equations:

(13) $$\begin{array}{c} D_{j}^{*}=\sum_{j=1}^{n}d\left({\tilde{v}}_{ij},{{\tilde{v}}_{i}}^{*}\right),\;j=1,2,\ldots ,J,\\ D_{j}^{-}=\sum_{j=1}^{n}d\left({\tilde{v}}_{ij},{{\tilde{v}}_{i}}^{-}\right),\;j=1,2,\ldots ,J. \end{array}$$

**Step 6**

Determine the similarities to ideal solution

(14) $$\begin{array}{c} CC_{j}^{*}=\frac{D_{j}^{-}}{D_{j}^{*}+D_{j}^{-}},\;j=1,2,\ldots ,J. \end{array}$$

**Step 7**

Rank the preference order.

## 3. The Proposed Model

The model proposed for the provider selection problem consists of two different kinds of fuzzy MCDM approaches: fuzzy AHP, which we used for calculating weights of criteria, and fuzzy TOPSIS for the ranking of alternative providers.

At first step, a decision making group is organized from experts, managers, and academics. Decision makers determined the selection criteria and provider alternatives, then they built the hierarchical structure of decision model.

After building the hierarchical structure, pairwise comparison matrix is established to identify the weights of criteria. The weights have been calculated by Chang's [21] extent analysis on fuzzy AHP based on previously determined linguistic variables by decision makers.

Finally, provider ranks have been determined by fuzzy TOPSIS in accordance with the linguistic variable values of providers. The alternative having the maximum *CC*_*j*_ value is selected as the most appropriate provider.

## 4. Illustrative Example

**Figure 1:**
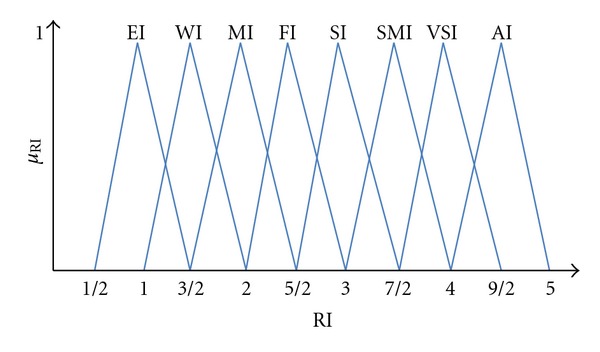
The linguistic scale of fuzzy triangular numbers.

Decision making group which is composed of experts, managers, and academics determined 6 important criteria out of 30 criteria. They eliminate the less important criteria in accordance with their experiments and knowledge. And the same group also determined 5 provider alternatives out of 15 firms. In order to take into account the uncertainty in judgements and vagueness in reasoning and by the help of membership functions we can exactly measure the perceptions. Therefore we used fuzzy linguistic variables applied.  Figure 1 shows the linguistic scale of fuzzy triangular numbers. 

### 4.1. Determination of Criteria Weights

**Figure 2:**
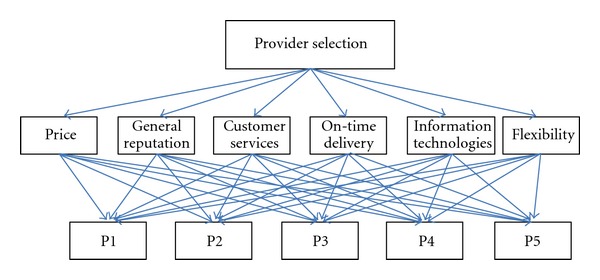
Hierarchical structure of the model.

Provider selection criteria in 3PL are decided as follows.  Figure 2 shows the hierarchical structure of the model.

1. Price (PR);
2. General reputation (GR);
3. Customer services (CS);
4. On-time delivery (OD);
5. Information technologies (IT);
6. Flexibility (FL).

Table 1 shows the Triangular fuzzy conversion scale of importance degrees and the pairwise comparison matrix of criteria is given in Table 2. After the calculations according to the pairwise comparison matrix in Table 3 through fuzzy AHP, the weights of criteria were determined.

### 4.2. Selection of the Provider

In this section we used the Chen's fuzzy linguistic scale as shown in Table 4 for calculations in fuzzy TOPSIS.

After the calculations according to Table 6 ranking of providers is determined. Fuzzy Evaluation Matrix for Providers is given on Table 5.

Same calculation steps are applied to all alternatives. Based on the *CC*_*j*_ values the maximum *CC*_*j*_ value is selected as the best provider (P3); after P3 the rank of alternatives in descending order is P5, P1, P4, and P2. 

## 5. Conclusions

Due to the rapid growth of industries and increased global competition, firms must take care of all processes of business. In order to enrich competitive advantages in market, firms are considering different strategies. Logistic outsourcing is one of these strategies. An effective provider selection plays a vital role both for outsourcing company and the provider. In general the necessary data for MCDM problems are imprecise and uncertain. Solving problems through fuzzy techniques eliminates the limitation of crisp values. The importance of the model is the vagueness of the subjective decision making, taken into account by using fuzzy techniques in fuzzy environment. More dependable, more sensitive, and more flexible results can be obtained through fuzzy approaches. Weights of provider selection criteria are determined through FAHP and providers ranked through fuzzy TOPSIS. This model integrates different fuzzy MCDMs in order to take advantages of different approaches. Owing to the hybrid structure the disadvantages of dependency to only one method is eliminated. The hybrid model aims to integrate the strong aspects of different fuzzy methods. 

Future researches may try to extend this study as an integration of more fuzzy MCDM techniques to solve many other decision making problems in many other disciplines. 

## Figures and Tables

**Table 1:** Triangular fuzzy conversion scale.

Linguistic scale for importance degrees	Triangular fuzzy scale	Triangular fuzzy reciprocal scale
Equally important	(1/2, 1, 3/2)	(2/3, 1, 2)
Weakly important	(1, 3/2, 2)	(1/2, 2/3, 1)
Moderately important	(3/2, 2, 5/2)	(2/5, 1/2, 2/3)
Fairly important	(2, 5/2, 3)	(1/3, 2/5, 1/2)
Strongly important	(5/2, 3, 7/2)	(2/7, 1/3, 2/5)
Strongly more important	(3, 7/2, 4)	(1/4, 2/7, 1/3)
Very strongly important	(7/2, 4, 9/2)	(2/9, 1/4, 2/7)
Absolutely important	(4, 9/2, 5)	(1/5, 2/9, 1/4)

**Table 2:** The pairwise comparison matrix of criteria.

	PR	GR	CS	OD	IT	FL
PR	—	MI	WI	EI	SI	VSI
GR		—		SMI		
CS		WI	—			
OD			SI	—	WI	SI
IT		SMI	MI		—	
FL		WI	EI		MI	—

**Table 3:** Weights of criteria.

*W*_PR_	0.293
*W*_GR_	0.101
*W*_CS_	0.038
*W*_OD_	0.299
*W*_IT_	0.19
*W*_FL_	0.079

**Table 4:** Chen's fuzzy scale.

Linguistic variable	Fuzzy scale
Very low (VL)	(0, 0, 0.1)
Low (L)	(0, 0.1, 0.3)
Medium low (ML)	(0.1, 0.3, 0.5)
Medium (M)	(0.3, 0.5, 0.7)
Medium high (MH)	(0.5, 0.7, 0.9)
High (H)	(0.7, 0.9, 1)
Very high (VH)	(0.9, 1, 1)

**Table 5:** Fuzzy evaluation matrix for providers.

	PR	GR	CS	OD	IT	FL
P1	M	ML	MH	MH	H	M
P2	H	MH	L	ML	MH	M
P3	VH	ML	ML	H	MH	H
P4	MH	ML	H	ML	MH	MH
P5	M	H	MH	H	ML	H

**Table 6:** Fuzzy TOPSIS results.

Alternatives	*D*_j_*	*D*_*j*_^−^	*CC*_*j*_	Ranking
P1	5.387	0.639	0.106	3
P2	5.411	0.616	0.102	5
P3	5.196	0.817	0.136	1
P4	5.398	0.629	0.104	4
P5	5.358	0.666	0.111	2
